# Prospective assessment using ^18^F-FDG PET/CT as a novel predictor for early response to PD-1 blockade in non-small-cell lung cancer

**DOI:** 10.1038/s41598-022-15964-3

**Published:** 2022-07-12

**Authors:** Ou Yamaguchi, Kyoichi Kaira, Ichiro Naruse, Yukihiro Umeda, Takeshi Honda, Satoshi Watanabe, Kosuke Ichikawa, Kazunari Tateishi, Norimitsu Kasahara, Tetsuya Higuchi, Kosuke Hashimoto, Shun Shinomiya, Yu Miura, Ayako Shiono, Atsuto Mouri, Hisao Imai, Kunihiko Iizuka, Tamotsu Ishizuka, Koichi Minato, Satoshi Suda, Hiroshi Kagamu, Keita Mori, Ichiei Kuji, Nobuhiko Seki

**Affiliations:** 1 Department of Respiratory Medicine, International Medical Center, Saitama Medical University, 1397-1 Yamane, Hidaka-City, Saitama 350-1298 Japan; 2 Department of Nuclear Medicine, International Medical Center, Saitama Medical University, 1397-1 Yamane, Hidaka-City, Saitama 350-1298 Japan; 3 Department of Respiratory Medicine, Hidaka Hospital, 886, Nakao-cho, Takasaki, 370-0001 Japan; 4 Cancer Center, Hidaka Hospital, 886, Nakao-cho, Takasaki, 370-0001 Japan; 5 Third Department of Internal Medicine, Faculty of Medical Sciences, University of Fukui, Matsuoka-Shimoaizuki, 23-3, Eiheiji, Fukui 910-1193 Japan; 6 Division of Medical Oncology, Department of Internal Medicine, Teikyo University School of Medicine, 2-11-1, Kaga, Itabashi-ku, Tokyo, 173-8606 Japan; 7 Department of Respiratory Medicine and Infectious Diseases, Niigata University Graduate School of Medical and Dental Sciences, 1-757 Asahimachidori, Chuouku, Niigata, 951-8510 Japan; 8 First Department of Internal Medicine, Shinshu University School of Medicine, 1-1-3, Asahi, Matsumoto-City, Nagano 390-8621 Japan; 9 Innovative Medical Research Center, Gunma University Hospital, Showa-machi, 3-39-15, Maebashi, Gunma 371-8511 Japan; 10 Department of Diagnostic Radiology and Nuclear Medicine, Gunma University Hospital, Showa-machi, 3-39-15, Maebashi, Gunma 371-8511 Japan; 11 Department of Internal Medicine, Public Tomioka General Hospital, 1-2073, Tomioka, Gunma 370-2316 Japan; 12 Division of Respiratory Medicine, Gunma Prefectural Cancer Center, 617-1, Takabayashinishi-cho, Ota, Gunma 373-8550 Japan; 13 Clinical Research Center, Shizuoka Cancer Center, 1007, Shimonagakubo, Sunto-gun, Shizuoka 411-8777 Japan

**Keywords:** Cancer, Oncology

## Abstract

Anti-programmed death-1 (PD-1) blockade is a standard treatment for advanced non-small-cell lung cancer (NSCLC). However, no appropriate modality exists for monitoring its therapeutic response immediately after initiation. Therefore, we aimed to elucidate the clinical relevance of ^18^F-FDG PET/CT versus CT in predicting the response to PD-1 blockade in the early phase. This prospective study included a total of 54 NSCLC patients. ^18^F-FDG PET/CT was performed at 4 weeks and 9 weeks after PD-1 blockade monotherapy. Maximum standardized uptake values (SUL_max_), metabolic tumor volume (MTV), and total lesion glycolysis (TLG) were evaluated. Among all patients, partial metabolic response and progressive metabolic disease after PD-1 blockade were observed in 35.2% and 11.1% on SUL_max_, 22.2% and 51.8% on MTV, and 27.8% and 46.3% on TLG, respectively, whereas a partial response (PR) and progressive disease (PD), respectively, based on RECIST v1.1 were recognized in 35.2% and 35.2%, respectively. The predictive probability of PR (MTV: 57.9% vs. 21.1%, *p* = 0.044; TLG: 63.2% vs. 21.1%, *p* = 0.020) and PD (MTV: 78.9% vs. 47.3%, *p* = 0.002; TLG: 73.7% vs. 21.1%, *p* = 0.007) detected based on RECIST at 4 weeks after PD-1 blockade initiation was significantly higher using MTV or TLG on ^18^F-FDG uptake than on CT. Multivariate analysis revealed that metabolic response by MTV or TLG at 4 weeks was an independent factor for response to PD-1 blockade treatment. Metabolic assessment by MTV or TLG was superior to morphological changes on CT for predicting the therapeutic response and survival at 4 weeks after PD-1 blockade.

## Introduction

Immune checkpoint inhibitors (ICIs) targeting programmed death-1 (PD-1)/PD ligand-1 (PD-L1) are widely used to treat human neoplasms. Recent studies on a limited population have shown that the therapeutic efficacy of ICIs was closely associated with long-term survival^1,2^. In particular, approximately 20% of patients with stage IV non-small-cell lung cancer (NSCLC) survived for > 5 years after PD-1 blockade monotherapy^1,2^. Approximately 80% of NSCLC patients exhibit a partial (PR) or complete (CR) response in the relatively early phase after PD-1 blockade initiation^1,2^. To establish the effectiveness of PD-1 blockade, its response should be detected as early as possible; however, it is challenging to distinguish responders from non-responders within approximately 9 weeks of treatment using computed tomography (CT) for morphological assessment^3,4^.

2-Deoxy-2-[fluorine-18]-fluoro-d-glucose (^18^F-FDG) positron emission tomography (PET)/ CT is useful for distinguishing benign from malignant lesions, and could predict early response to targeting agent in advanced NSCLC patients^5,6^F-FDG uptake within tumor cells is determined by the presence of increased glucose metabolism and hypoxia^5^, whereas upregulation of PD-L1 expression is partially regulated by tumor hypoxia^7,8^. Several studies have demonstrated that the accumulation level of ^18^F-FDG is closely associated with PD-L1 expression in NSCLC patients^9–12^.

Recently, it was proposed that ^18^F-FDG PET could predict the response to PD-1 blockade at the early phase within 2 or 4 weeks, whereas CT failed to detect early response^4,13^. However, previous reports were preliminary and could not confirm the therapeutic significance of ^18^F-FDG PET for early monitoring of ICI treatment. To date, no established biomarkers for predicting the efficacy of PD-1 blockade exist, although PD-L1 expression and tumor mutation burden (TMB) weakly correlate with the therapeutic efficacy of ICIs^14^. Although several devices, such as CT, have been used to evaluate tumor shrinkage, ^18^F-FDG PET has been identified as the best modality to reflect tumor metabolic changes in the early phase^4^. As the assessment metrics of ^18^F-FDG uptake, maximum standardized uptake values (SUL_max_), metabolic tumor volume (MTV), and total lesion glycolysis (TLG) are frequently used, with MTV or TLG suggested as better biomarkers reflecting the tumor burden than SUL_max_^15^.

Here, we aimed to elucidate the possibility of therapeutic monitoring using ^18^F-FDG PET at 4 and 9 weeks after PD-1 blockade in advanced NSCLC patients. Through a multi-institutional study using different PET scanners, we attempted to confirm whether ^18^F-FDG PET could be useful for detecting responders or non-responders in the early phase after ICI treatment.

## Results

### Patient characteristics

Fifty-eight patients were initially enrolled, but four were excluded because of inadequate PET study and data unavailability. A total of 54 patients (38 men, 26 women; median age, 73 years; age range, 42–84 years) were analyzed. Their demographics are presented in Table 1. Forty-two (77.8%) patients had a smoking history, and PS was 0, 1, and 2 in 12 (22.2%), 31 (57.4%), and 11 (20.4%) patients, respectively. Regarding the histological type, adenocarcinoma (AD) was observed in 29 (53.7%), squamous cell carcinoma (SCC) in 15 (27.8%), and other in 10 (18.5%). As treatment lines, 22 (40.7%) patients received pembrolizumab in first-line and 32 (59.3%) in second or more lines (nivolumab in 25 patients, pembrolizumab in 6, and atezolizumab in 1). Among the 54 patients, 19, 16, and 19 exhibited PR, stable disease (SD), and progressive disease (PD), respectively. The objective response was 35.2% (50.0% in first-line; 25.0% in second or more lines).

**Table 1:** Patient’s demographics.

Different variables		N = 54
Age	Median (range) years	73 years (42–84)
Gender	Male/Female	42/12
ECOG PS	0/1/2	12/31/11
Smoking	Yes/No	42/12
Histology	AD/SCC/Other	29/15/10
Disease stage	III/IV/Ope rec	3/39/10
T factor	T1/T2/T3/T4	5/14/14/11
N factor	N0/N1/N2/N3	9/7/7/21
M factor	M0/M1	4/40
Driver mutation	Wild/EGFR/ALK/unknown	44/6/1/3
PD-L1	< 1%/1–49%/50%↑/unknown	14/12/19/19
Treatment line	1st line/2nd or more line	22/32
PD-1 blockade	Nivo/Pemb/Atezo	25/28/1
Treatment response	PR/SD/PD	19/16/19

*ECOG* eastern cooperative oncology group, *PS* performance status, *AD* adenocarcinoma, *SCC* squamous cell carcinoma, *Ope rec* recurrence after operation, *PD-L1* programmed death ligand-1, *PD-1* programmed death-1, *EGFR* epidermal growth factor receptor, *ALK* anaplastic lymphpma kinase, *Nivo* nivolumab, *Pemb* pembrolizumab, *Atezo* atezolizumab, *PR* partial response, *SD* stable disease, *PD* progressive disease.

### PET study and response evaluation

There were 100 assessable target lesions, including 47 pulmonary, 37 metastatic lymph nodes, 6 pleural, 8 metastatic bone, 1 adrenal metastasis, and 1 liver metastasis. Median MTV, TLG, SUL_peak_, and SUL_max_ values before PD-1 blockade treatment were 19.2 (range, 2.8–95.2) cm^3^, 85.2 (range, 8.2–972.2) gcm^3^/mL, 8.3 (range, 3.9–17.0), and 8.6 (range, 2.5–22.7), respectively. The assessable lesion for SUL was defined as that with the highest ^18^F-FDG uptake. Baseline SUL_max_ values were significantly correlated with MTV (r = 0.357, *p* = 0.008) and TLG (r = 0.441, *p* < 0.001) values. The median period from baseline ^18^F-FDG PET/CT to PD-1 blockade monotherapy initiation was 9 (range, 1–28) days and that from PD-1 blockade monotherapy to first (4 weeks) and second (9 weeks) performance of ^18^F-FDG PET/CT was 28 and 63 days, respectively. Fifty-four patients underwent ^18^F-FDG PET/CT at 4 weeks; however, 6 did not receive ^18^F-FDG PET/CT at 9 weeks because of PD-1 blockade cessation due to PD and/or severe adverse events.

Among the 54 patients, PR, SD, and PD detected by early CT (4 weeks) were noted in 19 (35.2%), 16 (29.6%), and 19 (35.2%) patients, respectively. PMR, SMD, and PMD detected by early PET (4 weeks) were observed in 35.2% (19/54), 53.7% (29/54), and 11.1% (6/54), respectively, on SUL_max_; 27.8% (15/54), 62.9% (34/54), and 9.2% (5/54), respectively, on SUL_peak_; 22.2% (12/54), 25.9% (14/54), and 51.8% (28/54) on MTV; and 27.8% (15/54), 25.9% (14/54), and 46.3% (25/54) on TLG. On early CT scan, PR, SD, and PD were 9.3% (5/54), 70.3% (38/54), and 20.4% (11/54), respectively.

**Figure 1:**
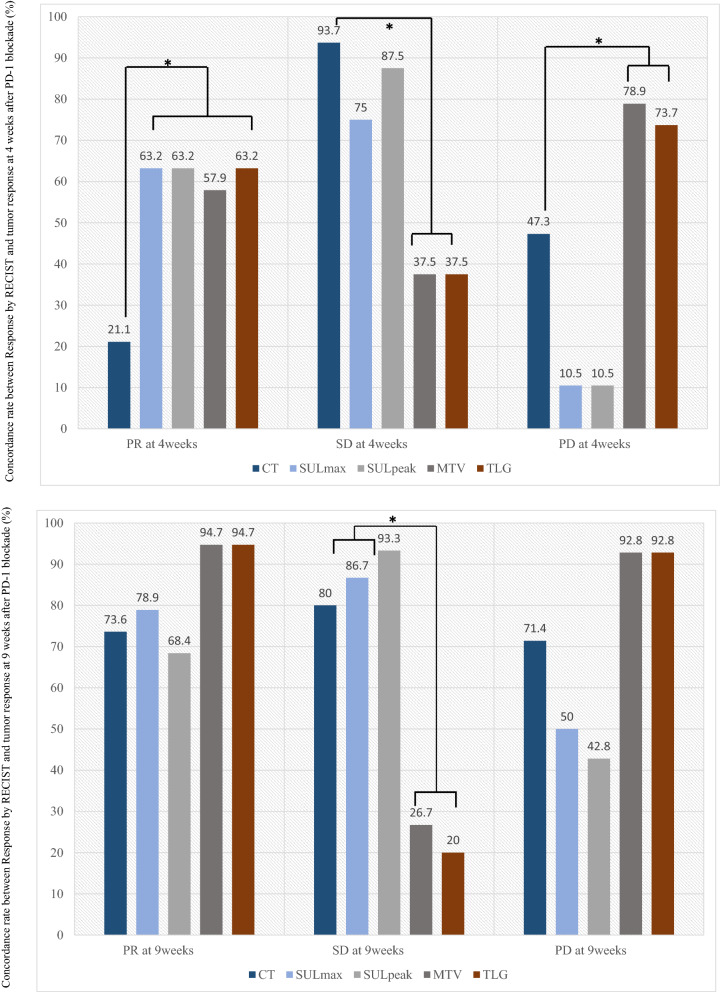
Concordance rate between Response by RECIST and tumor response at 4 weeks (**A**) and 9 weeks (**B**) after PD-1 blockade. (**A**) Among the 19 patients with PR based on RECIST, PMR at 4 weeks after PD-1 blockade by SUL_max_, SUL_peak_, MTV, and TLG was observed in 12 (63.2%), 12 (63.2%), 11 (57.9%), and 12 (63.2%), respectively, and CT at 4 weeks confirmed PR in 4 of 19 patients (21.1%). In the 19 patients with PD based on RECIST, PMD at 4 weeks by SUL_max_, SUL_peak_, MTV, and TLG was noted in 2 (10.5%), 2 (10.5%), 15 (78.9%), and 14 (73.7%), respectively, whereas CT at 4 weeks identified PD in 9 of 19 patients (47.3%). The predictive probability of PR and PD according to RECIST at 4 weeks after PD-1 blockade administration was significantly higher in MTV and TLG than in CT, whereas, the predictive probability of SD after its treatment was significantly higher in CT than in MTV and TLG. (**B**) Moreover, PMR at 9 weeks by SUL_max_, SUL_peak_, MTV, and TLG was observed in 15 (78.9%), 13 (68.4%), 18 (94.7%), and 18 (94.7%) of 19 patients with PR according to RECIST, respectively, and CT at 9 weeks confirmed PR in 10 of 14 patients (73.6%). Among the 19 patients with PD according to RECIST, PMD at 9 weeks by SUL_max_, SUL_peak_, MTV, and TLG was identified in 7 (50.0%), 6 (42.8%), 13 (92.8%), and 13 (92.8%), respectively, and CT at 9 weeks displayed PD in 10 of 14 patients (71.4%). The predictive probability of PR and PD according to RECIST at 9 weeks after PD-1 blockade administration was significantly higher in CT and SUL_max_ than in MTV and TLG. *Statistically significant difference.

The concordance rate of tumor response between confirmed overall objective response (OOR) by RECIST and that at 4 and 9 weeks after PD-1 blockade treatment are shown in Fig. 1. The concordance rate of PR (MTV: 57.9% vs. 21.1%, *p* = 0.044, TLG; 63.2% vs. 21.1%, *p* = 0.020) and PD (MTV: 78.9% vs. 47.3%, *p* = 0.002; TLG: 73.7% vs. 21.1%, *p* = 0.007) detected based on RECIST at 4 weeks after PD-1 blockade initiation was significantly higher in MTV or TLG than in CT (Fig. 1A). SUL_max_ and SUL_peak_ were significantly superior to CT in the concordance rate of PR at 4 weeks (63.2% vs. 21.1%, *p* = 0.020) whereas they were inferior to CT in the concordance rate of PD at 4 weeks (10.5% vs. 47.3%, *p* = 0.029) (Fig. 1A). Although the concordance rate of PR (94.7% vs. 73.6%, *p* = 0.179) and PD (92.8% vs. 71.4%, *p* = 0.325) confirmed based on RECIST at 9 weeks was higher in MTV or TLG than in CT, it was not statistically significant (Fig. 1B). Next, concordance rates according to treatment lines (first-line and second or more line settings) and histological type were also examined (Fig. C, online only). The concordance rate of PR and PD in the ^18^F-FDG uptake (SUL_max_, MTV and TLG) at 4 weeks tended to be high in patients with non-AC compared to those with AC, without statistical significance (Figs. C1, C3). Moreover, the concordance rate of PR in the ^18^F-FDG uptake (SUL_max_, MTV and TLG) at 4 weeks exhibited a significantly higher in first-line setting than in second line or more (Figs. C5, C7).

Out of 54 patients, 2 (3.7%) patients experienced pseudoprogression. One patient with confirmed PR based on RECIST, had PMD by MTV and TLG, PD by CT scan, and SMD by SUL_max_ and SUL_peak_ at 4 weeks after first-line pembrolizumab, because of markedly increased primary site. However, PMR was observed by MTV, TLG, SUV, and SUV on ^18^F-FDG PET at 9 weeks, similar to PR by CT scan. Although the other patient with confirmed PR based on RECIST also experienced psuedoprogression within 4 weeks after nivolumab initiation as second line setting, the objective response by CT at 4 and 9 weeks exhibited SD, that by MTV and TLG showed SMD at 4 weeks and PMR at 9 weeks, and that by SUL_max_ and SUL_peak_ depicted PMR at 4 and 9 weeks.

### Survival analysis according to ^18^F-FDG uptake

**Figure 2:**
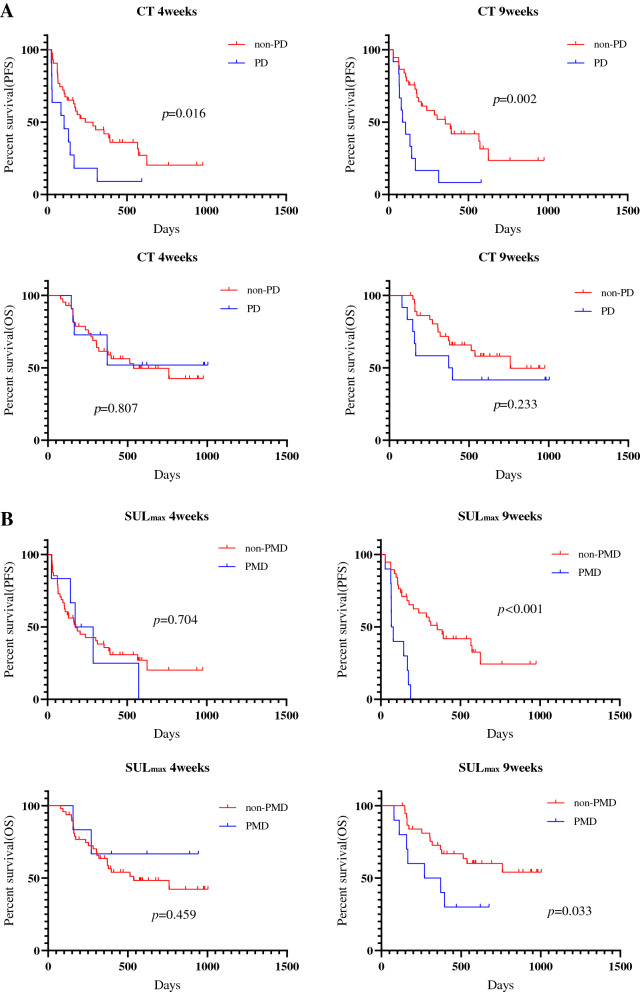

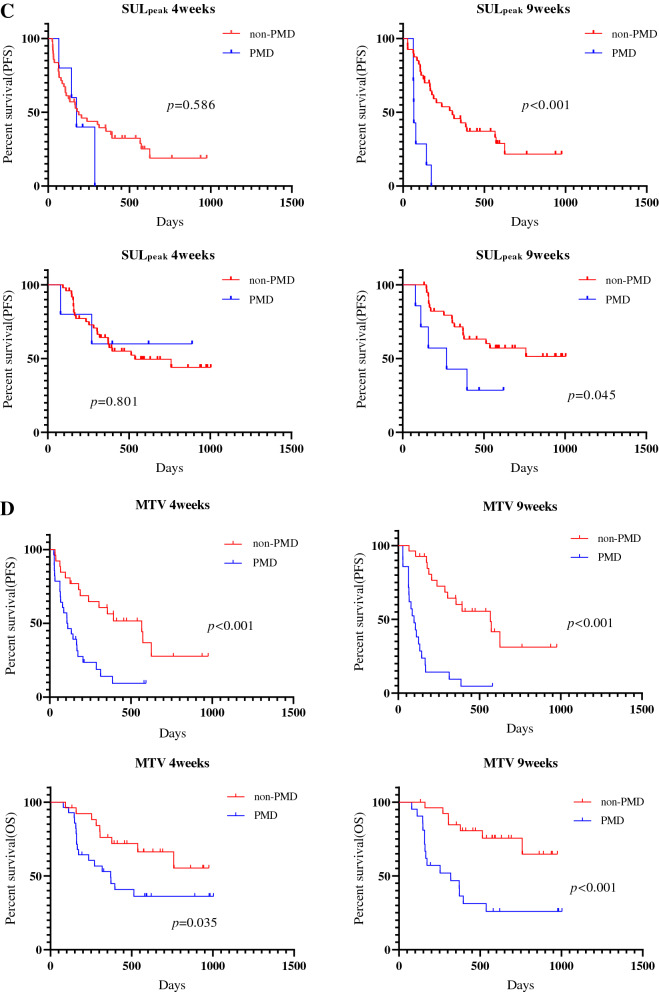

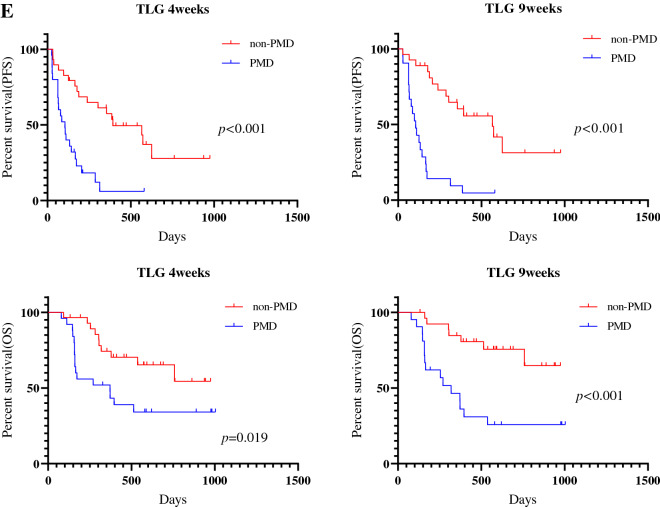
Kaplan–Meier curve of PFS and OS according to CT and ^18^F-FDG uptake at 4 and 9 weeks after PD-1 blockade initiation in all patients (n = 54). A significant difference in PFS, but not in OS, was noted between PD and non-PD defined according to CT at 4 and 9 weeks (**A**). A significant difference in PFS and OS was identified between PMD and non-PMD defined according to the ^18^F-FDG uptake by SUL_max_ (**B**) and SUL_peak_ (**C**) at 9 weeks, but not at 4 weeks. A significant difference in PFS and OS was identified between PMD and non-PMD defined according to ^18^F-FDG uptake by MTV (**D**) and TLG (**E**) at 4 and 9 weeks.

**Figure 3:**
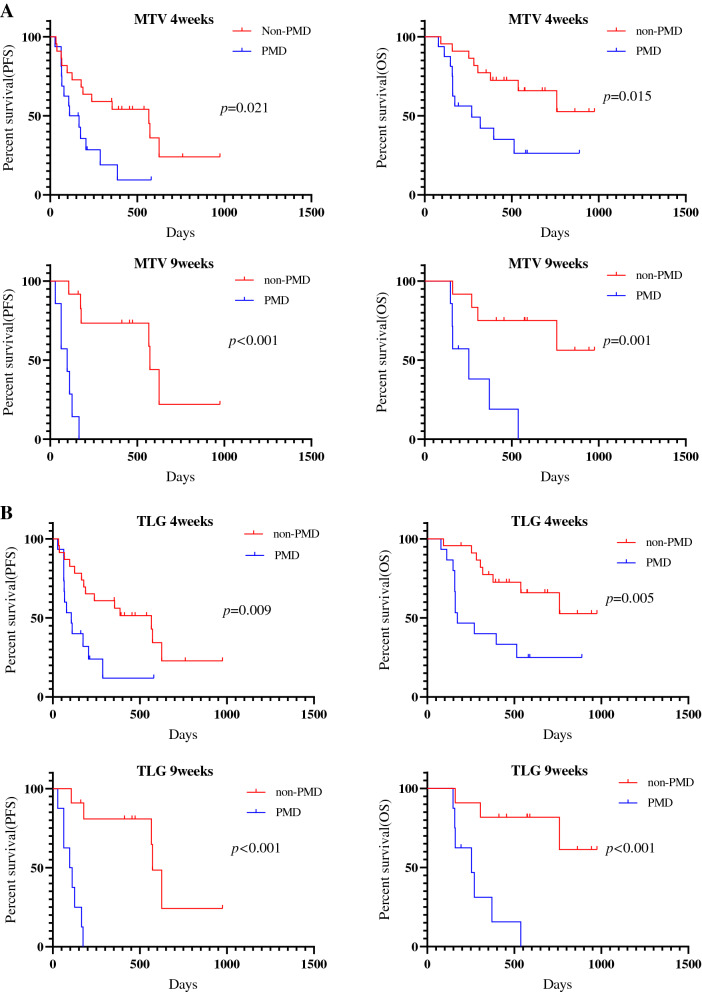

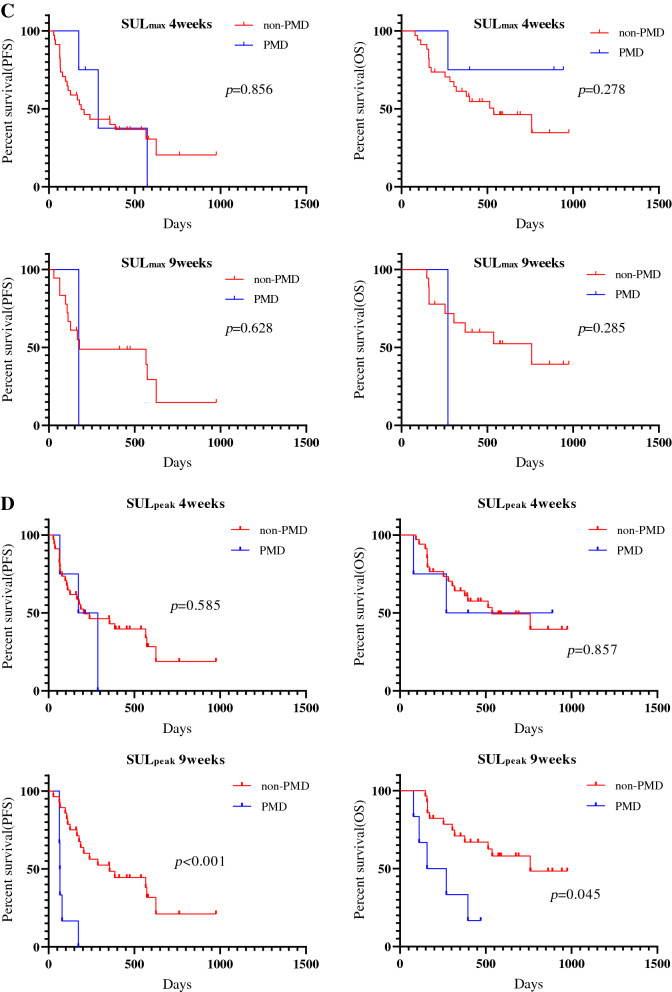
Kaplan–Meier curve of PFS and OS according to ^18^F-FDG uptake at 4 and 9 weeks in 38 patients with SD on CT scan at 4 weeks after PD-1 blockade initiation. A significant difference in PFS and OS was noted between PMD and non-PMD based on MTV (**A**) and TLG (**B**) at 4 and 9 weeks, but not SUL_max_ at 4 and 9 weeks (**C**). A significant difference in the PFS and OS according to SUL_peak_ at 9 weeks was observed between PMD and non-PMD, but no at 4 weeks (**D**).

The median follow-up period for all patients was 296 days (range 75–741). Forty patients experienced disease recurrence, and 21 died. The median PFS and OS were 174 days and not reached, respectively. Kaplan–Meier curves of PFS and OS according to CT and ^18^F-FDG uptake at 4 and 9 weeks after PD-1 blockade among all patients are shown in Fig. 2. A significant difference in PFS and OS was identified between PMD and non-PMD defined according to ^18^F-FDG uptake by MTV and TLG at 4 and 9 weeks, but not at 4 weeks but 9 weeks on PET by SUL_max_ (Fig. 2). Next, the outcome of 38 patients with SD on CT scan at 4 weeks after PD-1 blockade initiation were analyzed according to metabolic response by ^18^F-FDG uptake (PMD vs. non-PMD) (Fig. 3). A significant difference in PFS and OS was identified between PMD and non-PMD based on MTV (Fig. 3A) and TLG (Fig. 3B) at 4 and 9 weeks, but not on PET by SUL_max_ (Fig. 3C). Results of the univariate and multivariate analyses are presented in Table 2. In multivariate analysis, MTV and TLG on ^18^F-FDG uptake at 4 weeks after PD-1 blockade were confirmed as independent predictive factors.

**Table 2:** Univariate and multivariate survival analysis from PD-1 blockade initiation in all patients.

Variables	PFS						OS					
	Univariate analysis		Multivariate analysis focusing on MTV		Multivariate analysis focusing on TLG		Univariate analysis		Multivariate analysis focusing on MTV		Multivariate analysis focusing on TLG	
	Median PFS (days)	*p* Value	HR (95% CI)	*p* Value	HR (95% CI)	*p* Value	Median PFS (days)	*p* Value	HR (95% CI)	*p* Value	HR (95% CI)	*p* Value
Age			1.006		1.222				1.115		1.303	
≦ 73/ > 73 years	168**/**178	0.561	(0.744–1.524)	0.727	(0.828–1.181)	0.311	173**/**270	0.931	(0.689–1.803)	0.655	(0.786–2.185)	0.303
Gender			0.753		0.857				0.736		0.718	
Male/female	174**/**188	0.869	(0.478–1.158)	0.201	(0.5392–1.344)	0.505	396**/**NR	0.512	(0.401–1.354)	0.315	(0.372–1.317)	0.291
ECOG PS			1.299		1.427				1.483		1.792	
0–1/2	178**/**65	0.225	(0.823–1.976)	0.249	(0.899–2.184)	0.126	NR**/**282	0.052	(0.880–2.501)	0.148	(1.022–3.038)	**0.042**
Smoking												
Yes/No	205**/**119	0.146					NR**/**NR	0.955				
Histology												
Ad/Non-Ad B	178**/**174	0.858					NR**/**396	0.763				
Treatment line												
1st/2nd or more	239**/**106	0.249					NR**/**396	0.409				
CT response at 4 W			0.779		0.761				1.216		1.242	
PD/Non-PD	105**/**188	**0.009**	(0.495–1.244)	0.290	(0.492–1.204)	0.235	NR**/**NR	0.913	(0.644–2.293)	0.538	(0.692–2.449)	0.499
Response by SUL_max_ at 4 W												
PMD/Non-PMD	174**/**178	0.997					NR**/**396	0.781				
Response by SUL_peak_ at 4 W												
PMD/Non-PMD	174**/**188	0.586					NR**/**537	0.806				
Response by MTV at 4 W			0.536						0.530			
PMD/Non-PMD	106**/**393	** < 0.0001**	(0.898–1.244)	**0.005**			325**/**NR	**0.018**	(0.308–0.911)	**0.019**		
Response by TLG at 4 W					0.477						0.453	
PMD/Non-PMD	105**/**386	** < 0.0001**			(0.307–0.732)	** < 0.001**	270**/**NR	**0.011**			(0.261–0.765)	**0.003**

*ECOG PS* eastern clinical oncology group performance status, *BI* brinkman index, *Ad* adenocarcinoma, *Non-Ad* non-adenocarcinoma, *ICI* immune checkpoint inhibitor, *irAEs* immune related adverse events, *PMD* progressive metabolic disease, *CT* computed tomography, *SUV*_*max*_ maximum of standardized under value, *MTV* metabolic tumor volume, *TLG* total lesion glycolysis, *4 W* 4 weeks after PD-1 blockade initiation.

Significant values are in bold.

## Discussion

This prospective study compared CT from PET for the assessment of early response after PD-1 blockade monotherapy in advanced NSCLC patients. The concordance rate of PR and PD using RECIST at 4 weeks after PD-1 blockade was significantly higher using ^18^F-FDG uptake by MTV or TLG than by using morphological changes on CT. In addition, PMD by MTV or TLG at 4 weeks could significantly predict worse survival after PD-1 blockade administration. At least 9 weeks after its administration, ^18^F-FDG uptake by MTV or TLG accurately predicted the tumor response confirmed based on RECIST and survival after PD-1 blockade, compared to the morphological assessment by CT. In this study, we also found that the concordance rate of PR and PD detected based on RECIST at early phase after PD-1 blockade tended to be higher in patients with non-AC or first-line setting. In particular, metabolic response by MTV and TLG could differentiate responder from non-responder in 38 patients with SD on CT at 4 weeks after PD-1 blockade administration.

Recently, Park et al.^16^ retrospectively evaluated early response assessment after immunotherapy using ^18^F-FDG PET/CT in 24 advanced NSCLC patients. They presented the case of 5 patients with CMR or PMR who had a clinical benefit after two or three cycles of ICI treatment, whereas none of the 14 patients with PMD experienced any clinical benefit^16^. It was speculated that 5 patients with SMD needed meticulous follow-up because of varying clinical benefits^16^. Castello et al.^17^ prospectively compared morphological and metabolic responses at 8 or 9 weeks after PD-1 blockade using ^18^F-FDG PET/CT in 35 NSCLC patients. Although they assessed the metabolic response by the SUV value, 3 (75%) of 4 patients with PR had PMR or CMR, 14 (87%) of 16 patients with PD exhibited PMD, and SMD was observed in 4 (26%) of 15 patients with SD^17^. In patients with SD on CT, metabolic response by PET could discriminate those with longer survival^17^. ^18^F-FDG uptake based on PERCIST or immunotherapy-modified PERCIST accurately reflects the overall metabolic response and survival after PD-1 immunotherapy in NSCLC patients^4,14,18^.

Currently, PD-L1 is considered a rough biomarker for the therapeutic prediction of PD-1 blockade, and promising markers such as TMB or tumor-infiltrating lymphocytes fail to identify the progression to ICI treatment. Thus, the presence of responders and their progression should be identified as early as possible after PD-1 blockade to predict long-term responders. In our study, ^18^F-FDG uptake on PET yielded a higher predictive value to achieve PR at 4 weeks in first-line setting or histology with non-AC compared to CT. However, it remains unclear about its detailed mechanisms, thus, further investigation is warranted to elucidate the results of our study by using large –sample size.

Rossi et al.^19^ compared between ^18^F-FDG and CT-based criteria as response assessment at 8 weeks after nivolumab in 48 NSCLC patients and reported a low overall concordance between CT-based and PET-based responses, but PMR assessed by PET predicted longer OS than CT-based PR. In their study, neither MTV nor TLG but SUV_peak_ was used to assess the metabolic response, and early response was not investigated, as we did in this study. Our results indicated that the concordance rate by SUL_max_ was apparently inferior to that by MTV or TLG, and there was no significant difference between metabolic response by SUL_max_ and morphological response by CT. However, MTV or TLG was identified as a significant marker to predict the tumor response and survival in the early phase, such as 4 weeks after PD-1 blockade, compared to CT. Thus, SUL_max_ on PET may not be suitable for early detection of ICI response. The present study includes the patients receiving PD-1 blockade as first-line and second-line or more setting, however, previous investigations discussed the therapeutic monitoring of ^18^F-FDG PET after immunotherapy in patients with previously treated NSCLC^4,13,16^. The added value of our study demonstrated that the tumor response detected by early PET is useful for the therapeutic prediction of first-line pembrolizumab, moreover, the therapeutic significance of monitoring by early PET is also different according to the histological types of NSCLC. Nowadays, PD-1 blockade is generally established treatment as first-line setting against the advanced NSCLC without any drive gene mutations. Compared to previous studies reporting the monitoring by PET after ICIs administration, the data of first-line pembrolizumab in our study is helpful for our clinical practice. Moreover, our analysis was on a per patient basis and not on a per lesion basis. As several lesions in the same patients may depict different ^18^F-FDG uptake, the therapeutic monitoring by ^18^F-FDG uptake based on a per patient basis would be helpful for evaluating the response and survival of ICIs treatment.

Although several investigations demonstrated that baseline ^18^F-FDG PET could predict the outcome of PD-1 immunotherapy in NSCLC patients, it is difficult to discriminate responders from non-responders or PD from non-PD patients by baseline ^18^F-FDG uptake^20–22^. Because ^18^F-FDG uptake was assessed according to different PET machines in individual institutions, it may not be consistent at baseline. Therefore, we did not explore the efficacy and survival of PD-1 blockade based on baseline ^18^F-FDG uptake.

Recently, Lopci et al.^23^ described the new guidelines of ^18^F-FDG PET imaging during immunotherapy treatment in patients with solid tumors. To identify pseudoprogressive patients after immunotherapy, the refinement of standard response evaluation guidelines is needed, thus, immune-related response criteria (irRC), immune RECIST (iRECIST) and immune-modified RECIST (irRECIST) for solid tumors were proposed^23^. However, there are no established immunotherapy guidelines to categorize the therapeutic response of patients with pseudoprogressive disease (PPD). The interpretation of ^18^F-FDG PET should take into account PPD during ICIs treatment. In the current study, we found two patients with PPD (3.7%; 2 of 54 patients). SUL_max_ and SUL_peak_ were useful to detect the exact response at 4 weeks after ICIs administration in one of 2 patients with PPD, whereas, it was difficult to identify the correct response at early phase by MTV and TLG on PET. In these two patients, ^18^F-FDG PET imaging could detect the true response based on RECIST at 9 weeks after PD-1 blockade administration. However, it remains unclear whether ^18^F-FDG uptake on PET could be helpful for the detection of PPD. In our study, the response evaluation by PET was investigated based on the RECIST, however, there are several new immunotherapy guidelines such as irRC, iRECIST and irRECIST. Although we tried to analyze our data using iRECIST, the results of response evaluation after immunotherapy were not different between RECIST and iRECIST (data not shown). Therefore, further investigation is warranted to establish a new response guideline during immunotherapy aside from the current guidelines.

There are several limitations in our studies. In this study, firstly, the SULs were not harmonized between the PET scanners. However, as these were devices of almost the same generation of the same manufacturer, we believe that changes before and after treatment of SULs, MTV, and TLG were captured to some extent in each facility. Our results warrant for large-scale multi-center research on harmonizing SULs of each PET scanner. Second, our sample includes heterogeneous populations such as AC and non-AC or first-line and second-line settings. As the concordance rate of objective response was different according to histology and treatment lines, further study should be focused on the selected patients. In the present study, 6 (20.6%) of 29 patients with AC yielded positive epidermal growth factor (*EGFR*) mutation and one patients had anaplastic lymphoma kinase (*ALK*)-echinoderm microtubule-associated protein-like 4 (*EML4*) fusion gene. Five of 7 patients with these driver mutations were confirmed as PD in second-line setting (data not shown). The AC patients harboring *EGFR* mutations are low sensitive to ICs and were identified as lower ^18^F-FDG uptake on PET than wild-type.^24^ This may bias the different concordance rate between AC and non-AC. Finally, it is unknown if morphological assessment with RECIST is suitable modality to evaluate response to ICI therapy^4,13^. The aim of our study is to compare PET with CT in the assessment of tumor response at early phase after PD-1 blockade initiation. However, we believe that metabolic tumor response by PET may be better to predict the response and outcome of ICIs compared to CT. Moreover, the optimal detection by CT scan in PPD after ICIs initiation is difficult, but, the therapeutic monitoring by ^18^F-FDG uptake on PET also seemed to yield some limitations for the early detection of PPD. Further study is warranted to develop the modality to confirm the presence of PPD at early phase after ICIs initiation.

In conclusion, metabolic assessment by MTV or TLG was useful in predicting an early therapeutic response and survival after PD-1 blockade, compared to morphological changes on CT, specially, in patients with non-AC or first-line setting. ^18^F-FDG uptake may be a promising biomarker for predicting the therapeutic efficacy of ICIs; thus, it may contribute to individualized treatment planning in clinical practice.

## Methods

### Patients

This prospective study enrolled advanced NSCLC patients who received PD-1 blockade monotherapy at multiple institutions between January 2019 and October 2020. The inclusion criteria were (a) pathologically confirmed NSCLC; (b) candidate for PD-1 blockade monotherapy such as nivolumab, pembrolizumab or atezolizumab in first-, second- or more lines; (c) performance status (PS) on the Eastern Cooperative Oncology Group of 0–2; (d) ^18^F-FDG PET/CT imaging scheduled within 4 weeks before the first cycle of PD-1 blockade monotherapy, and (e) possessing adequate organ functions. The exclusion criteria were (a) evidence of concurrent cancer, (b) uncontrolled diabetes mellitus, (c) interstitial pneumonia or pulmonary fibrosis, and (d) active infection requiring antibiotic therapy. Baseline ^18^F-FDG PET/CT was performed as part of the disease evaluation workup before initiating PD-1 blockade monotherapy. Post-treatment PET/CT was needed at 4 and 9 weeks after the first cycle of PD-1 blockade. The protocol required that both pre- and post-treatment PET-CT be performed using the same scanner (Fig. A, online only).

This study was approved by the institutional review board (Saitama Medical University) and conducted according to the Declaration of Helsinki. All patients provided written informed consent before participation and were able to withdraw from the study at any time. This trial was registered in Japan Registry of Clinical Trials (jRCTs031180036) on 01/11/2018.

### PET imaging and data analysis

Patients fasted for at least 6 h before ^18^F-FDG administration for PET, performed using a PET/CT scanner. Three-dimensional data acquisition was initiated 60.0 ± 8.1 min after FDG injection. Attenuation-corrected transverse images obtained with ^18^F-FDG were reconstructed with the conditions of clinical settings at the institutions. Imaging parameter for PET/CT scanner at different institutions are listed in Table A1 (online only).

For semiquantitative analysis, the standardized uptake value (SUV) in lean body mass (LBM) corrected for the Japanese population was obtained based on the injected dosage of ^18^F-FDG, patient’s bodyweight, and cross-calibration factor between PET and the dose calibrator. SUV correction by LBM was based on the literature^25^. The SUL and Japanese-corrected LBM (JLBM) are defined as follows:

$$ {\text{SUL}} = {\text{radioactive}}\;{\text{concentration}}\;{\text{in}}\;{\text{the}}\;{\text{volume}}\;{\text{of}}\;{\text{interest}}\left( {{\text{VOI}}} \right)\left( {{\text{MBq}}/{\text{g}}} \right)/{\text{injected}}\;{\text{dose}}\left( {{\text{MBq}}} \right)/{\text{patient's}}\;{\text{LBM}}\left( {\text{g}} \right). $$

$$ {\text{JLBM}}\;{\text{in}}\;{\text{males}} = 28.27 \times {\text{height}}\left( {\text{m}} \right) + 0.359 \times {\text{weight}}\left( {{\text{kg}}} \right) - 0.032 \times {\text{age}}\left( {\text{y}} \right) - 21.83 $$

$$ {\text{JLBM}}\;{\text{in}}\;{\text{females}} = 26.12 \times {\text{height}}\left( {\text{m}} \right) + 0.253 \times {\text{weight}}\left( {{\text{kg}}} \right) - 0.022 \times {\text{age}}\left( {\text{y}} \right) - 19.58 $$

CT for initial staging was performed with an intravenous contrast medium, and board-certified radiologists interpreted the images. We used RAVAT software (Nihon Medi-physics Co. Ltd., Japan) on a Windows workstation to semi-automatically calculate the maximum SUL (SUL_max_), SUL_peak_, MTV, and TLG, defined as MTV multiplied by SUL_mean_, of each lesion using SUL thresholds obtained by the SUL in the liver VOI. Each threshold was defined as the average of 1.5 × SUL (SUL_mean_) plus 2 × SD of SUL in the liver^26,27^. These SUL thresholds were the optimum values for generating a 3D VOI in which the whole tumor mass was completely enclosed in all cases using the CT image as the reference. Regions of activity other than tumors, including myocardium, gastro-intestinal tracts, kidneys, and urinary tracts, were eliminated manually according to the orientation provided by the board-certified nuclear medicine physician.

In this study, SULs between facilities and between devices were not harmonized.

### Efficacy assessment

The confirmed tumor response was assessed according to the findings in CT imaging at 9 weeks interpreted using the Response Evaluation Criteria in Solid Tumors version 1.1 (RECIST v. 1.1). PET-based tumor response was defined according to the PET Evaluation Criteria in Solid Tumors (PERCIST) guidelines^27^: complete metabolic response (CMR), complete resolution of ^18^F-FDG uptake within the target lesion; partial metabolic response (PMR), ≥ 30% decrease in ^18^F-FDG uptake in the target tumor; progressive metabolic disease (PMD), ≥ 30% increase in ^18^F-FDG uptake in the target tumor or the advent of new ^18^F-FDG avid lesions; stable metabolic disease (SMD), neither CMR, PMR, nor PMD.

### Statistical analysis

Statistical analyses were performed using Student’s *t-*test and χ^2^ test for continuous and categorical variables, respectively. Statistical significance was set at *p* < 0.05. Correlations between SUL_max_, MTV, and TLG on ^18^F-FDG uptake were analyzed using Pearson’s rank test. Univariate and multivariate analyses of the relationship between scoring by ^18^F-FDG uptake and different variables were performed using logistic regression analysis. Progression-free survival (PFS) was defined as the time from initial immunotherapy to disease progression or death; OS was defined as the time from initial immunotherapy to death from any cause. The Kaplan–Meier method was used to estimate survival as a function of time, and survival differences were analyzed using log-rank test. The progression of disease for survival analysis was defined as progression in imaging.

Metabolic responses at 4 and 9 weeks after the injection of PD-1 blockade were evaluated according to the response on PET^25^. All statistical analyses were performed using GraphPad Prism software (v.8.0; GraphPad Software, San Diego, CA, USA) and JMP 14.0 (SAS Institute Inc., Cary, North Carolina, USA).

## Supplementary Information

Supplementary Information 1.

Supplementary Information 2.

Supplementary Information 3.

## Data Availability

The datasets used and/or analysed during the current study available from the corresponding author on reasonable request.
