# Supplementary material for: Prospective assessment using 18F-FDG PET/CT as a novel predictor for early response to PD-1 blockade in non-small-cell lung cancer
Source: Sci Rep. 2022 Jul 12;12:11832. doi: 10.1038/s41598-022-15964-3 (PMC9276827; doi:10.1038/s41598-022-15964-3)
Supplement: Supplementary file 1 — Supplementary Information 1. [file 41598_2022_15964_MOESM1_ESM.docx]

**Table A1. Imaging parameters of PET/CT scanners in different institutions**

| Institution | PET scanners | Field of view | Injection dose (FDG) | Uptake time (min) | Acquisition time | Image reconstruction method | Image matrics | Slice thickness | CT detectors | CT radiation dose (mAs) |
| --- | --- | --- | --- | --- | --- | --- | --- | --- | --- | --- |
| Saitama Medical Hospital | Siemens biograph 6/16 | 585mm | 3.7MBq/kg | 60 min. | 2min/bed | 3D-OSEM (FORE)  (iteration 3, subset 8) | 168ｘ168 | 2mm | 6/16 | Care Dose 50mAs |
| Gunma University Hospital | GE discovery ST Elite | 600mm | 5MBq/kg | 60 min. | 2min/bed | 3D-OSEM (FORE)  (iteration 3, su  bset 8) | 128ｘ128 | 3.75mm | 16 | Auto mA |
| Niigata University Hospital | Siemens  Biograph mCT　Flow 20 | 815 mm | 2-4 MBq/kg  (delivery system; full dose of 185MBq vial) | 60 min  ±10min | Flow motion (variable according to body size and dosage) | 3D-OSEM  (iteration:2,subset:21,Gaussian filter:5mm) +TOF | 200 x 200 | 2mm | 16/20 | Care Dose 80mAs |
| Hidaka Hospital | Aquiduo | 585mm | 4.0MBq/kg | 60 min. | 2min/bed | FORE+Aw-OSEM  (iteration4, sbset14) | 128ｘ128 | 2mm | 16 | Care Dose (Volume EC) |
| Shinshu University Hospital | Siemens  Biograph40mCT | 550mm | 3.9MBq/kg | 60 min. | 3min/bed | 3D-OSEM(TrueX＋TOF(ultraHD-PET))  (iteration 3, subset 21) | 200ｘ200 | 3mm | 40 | Care Dose 4D 70mAs |
| Teikyo University Hospital | Siemens Biograph 40 | 500 mm | Delivery | 60 min | 2 min/bed | Iterative 3D | 168ｘ168 | 5 mm | 40 | Care Dose (Auto) |
| Tomioka General Hospital | Siemens | 700mm | 3.8MBq/kg | 60 min. | 2min/bed | TrueX+TOF(UltraHD-PT) (iteration 3, subset 10) | 512 x 512 | 5mm | 16 x 1.2mm | Care Dose 82mAs |
| Gunma cancer center | SIEMENS  Biograph Vision450 | 350mm | 276.5±14.7  MBq | 60 min. | 0.9mm/sec  (CBM* bed  speed) | True+TOF  (ultraHD-PET)  (iteration4 Subsets5) | 440x440 | 2mm | 64 | CARE Dose4D  (Quality ref. mAs 220mAs) |
| Fukui University Hospital | Siemens  Biograph40　mCT Flow | 780mm | Ave: 4.27MBq/kg  Min: 3.77MBq/kg  Max: 4.63MBq/kg | 60 min. | Speed:1.2mm/s  Ave: 13.23min  Min: 12.64min  Max: 13.88min | 3D-OSEM (iteration 3, subset 21)（TOF＋PSF） | 200 x 200 | 4mm | 40 | Care Dose 4D  Eff. mAs:20 |
