# Supplementary material for: Prospective assessment using 18F-FDG PET/CT as a novel predictor for early response to PD-1 blockade in non-small-cell lung cancer
Source: Sci Rep. 2022 Jul 12;12:11832. doi: 10.1038/s41598-022-15964-3 (PMC9276827; doi:10.1038/s41598-022-15964-3)
Supplement: Supplementary file 2 — Supplementary Information 2. [file 41598_2022_15964_MOESM2_ESM.pdf]

Schedule of study

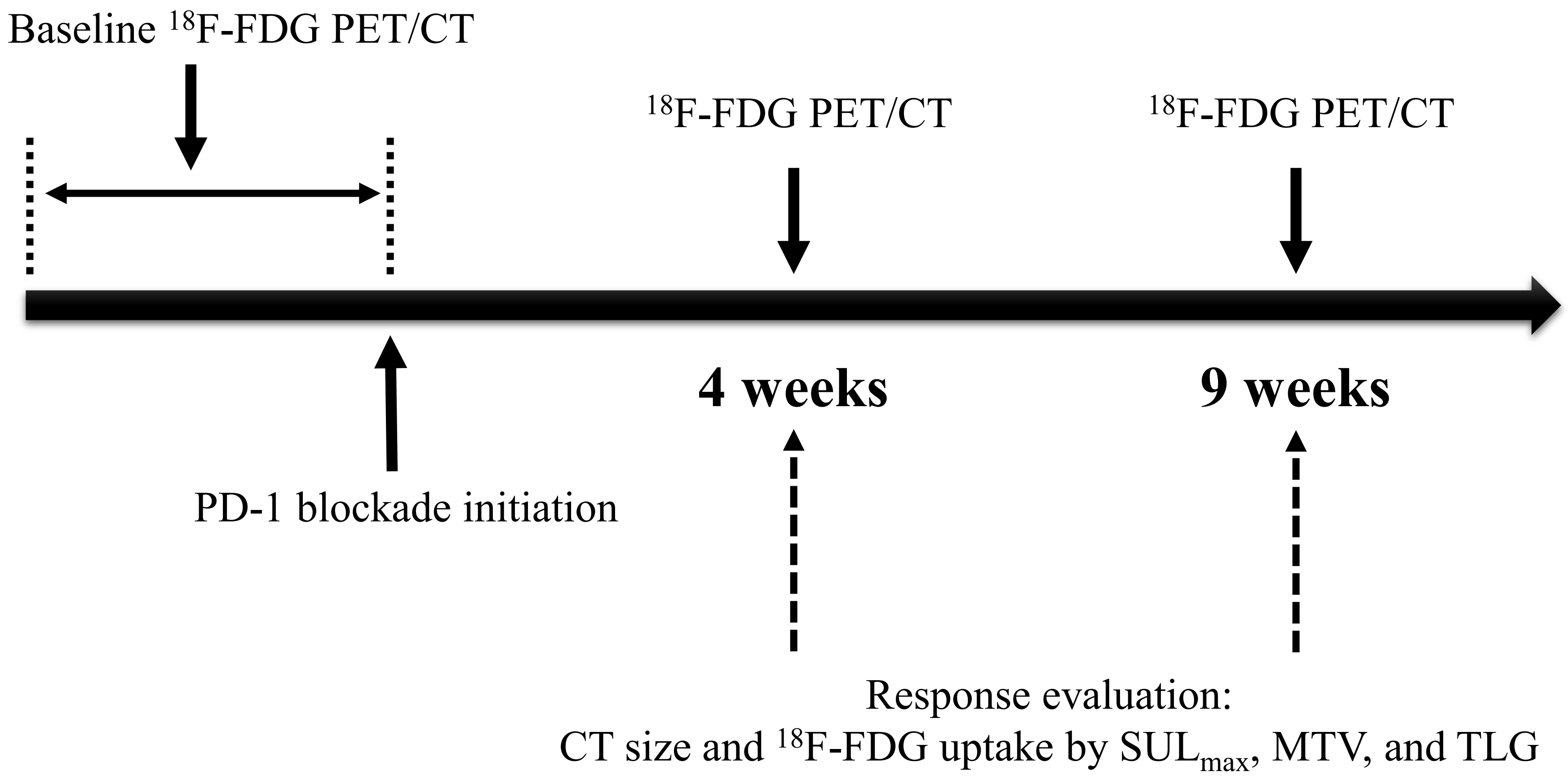

Figure legend: Schedule of ^{18}F-FDG PET/CT imaging in the present study:
^{18}F-FDG PET/CT imaging at baseline is performed within 30 days of study registration. Second and third PETs are planned to be performed at 4 and 9 weeks after PD-1 blockade monotherapy.

Figure A

Progression-free survival and Overall survival according to objective response

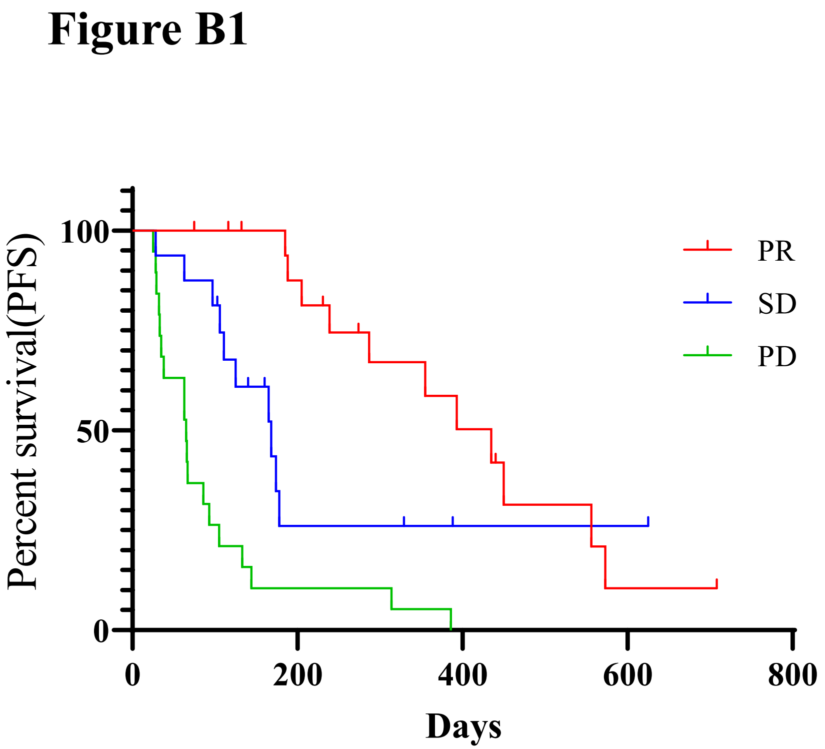

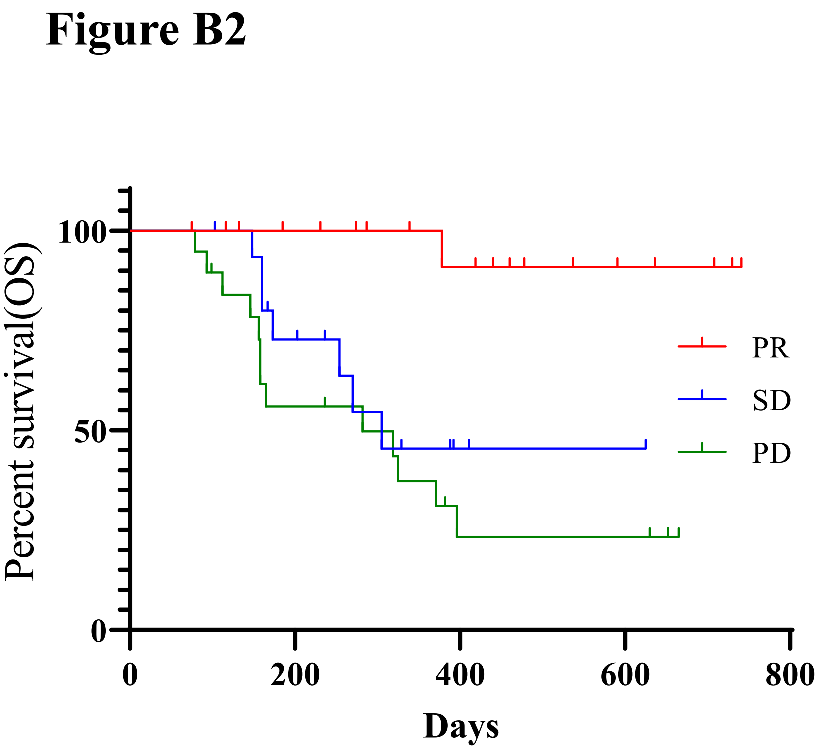

Figure legend: Progression-free survival (PFS) (B**1**) and overall survival (OS) (**B2**) according to objective response confirmed based on RECIST.

The median PFS and OS of PR, SD, and PD were 435, 168, and 65 days, respectively (PR vs. SD, p=0.029; SD vs. PD, p=0.001), and not reached, 305, and 282 days, respectively (PR vs. SD, p=0.004; SD vs. PD, p=0.276).

Figure B

Concordance rate in CT and PET

Legends: concordance rate at 4 weeks in patients with adenocarcinoma

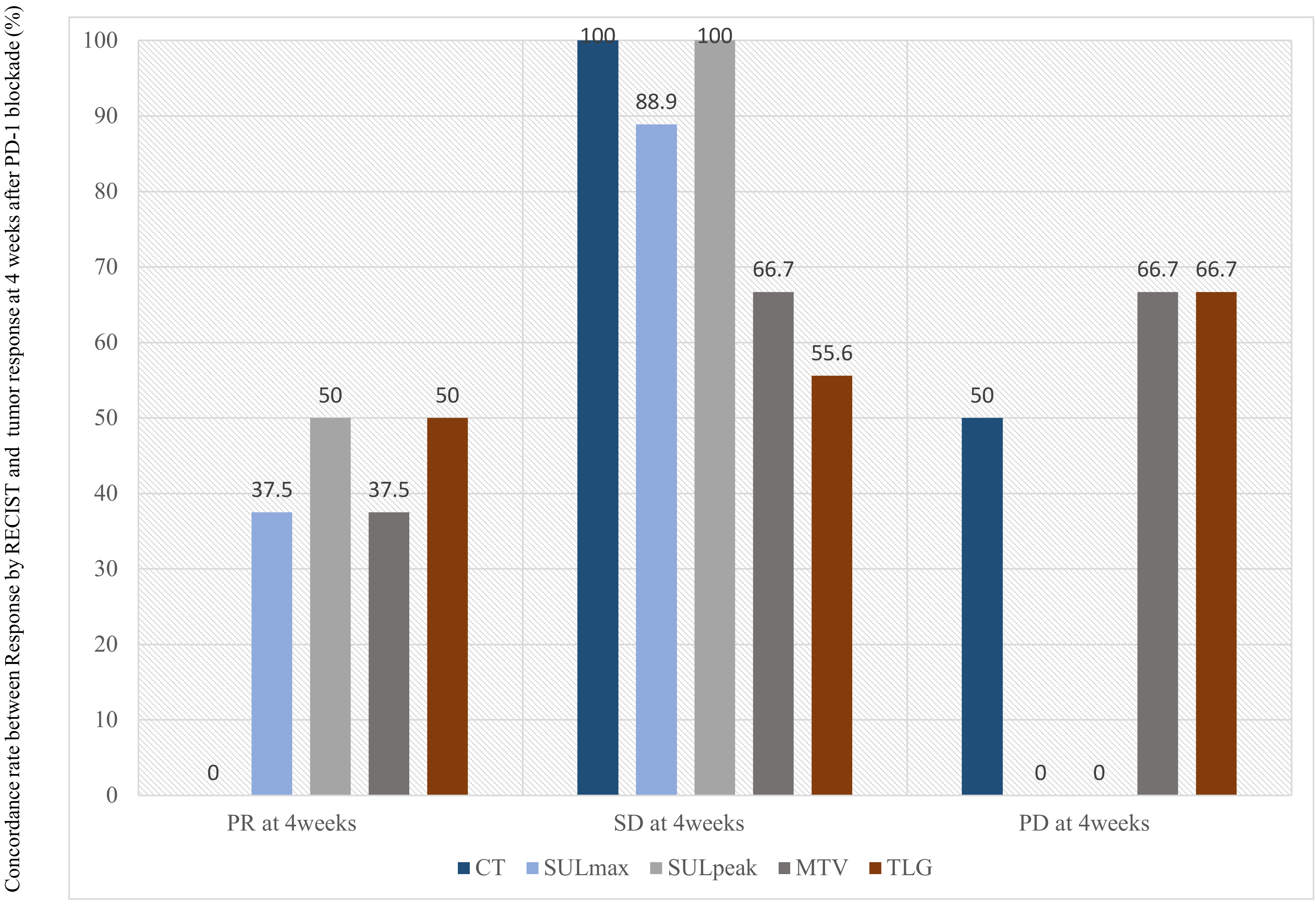

Figure C1

Concordance rate in CT and PET

Legends: concordance rate at 9 weeks in patients with adenocarcinoma

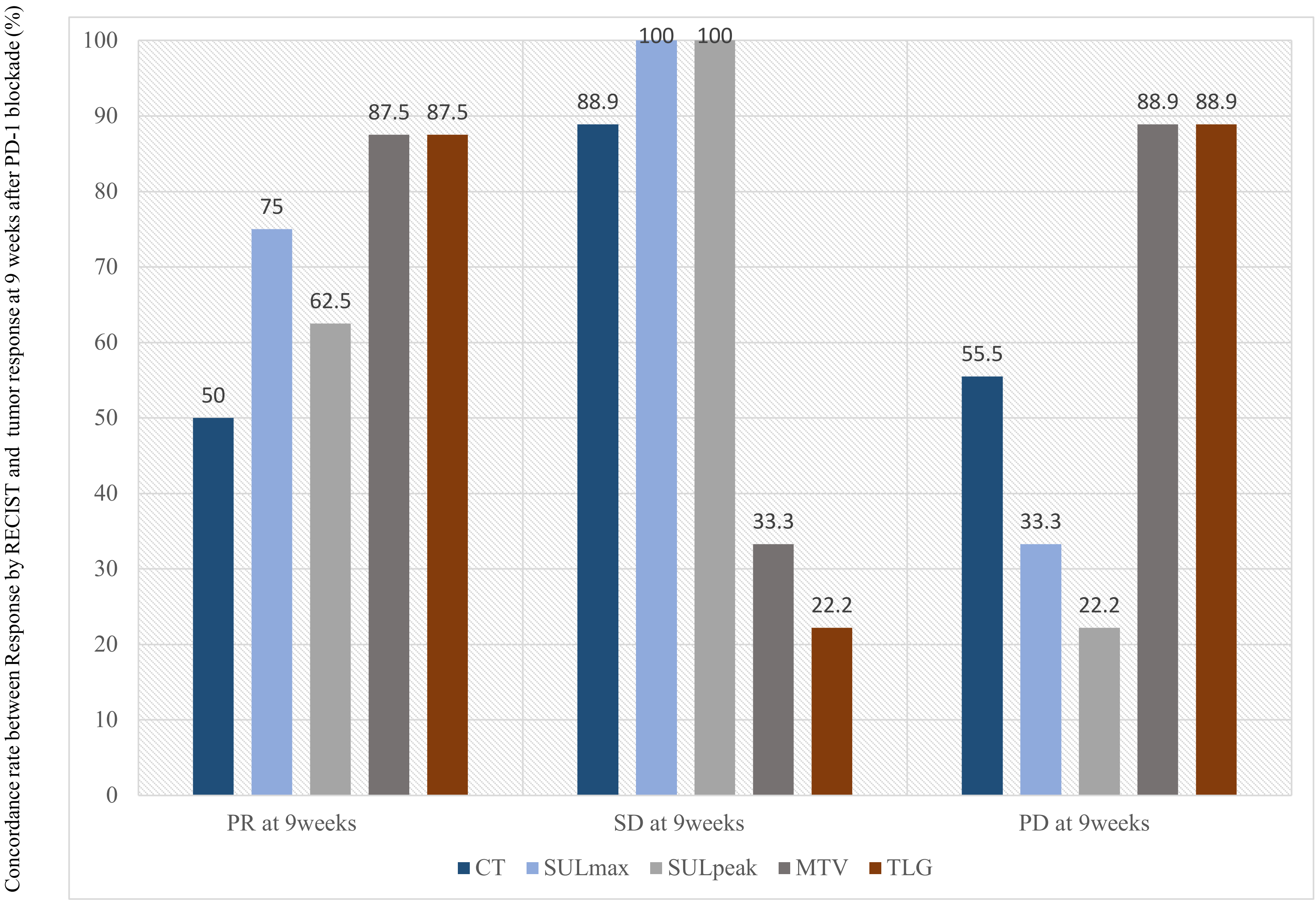

Figure C2

Concordance rate in CT and PET

Legends: concordance rate at 4 weeks in patients with non-adenocarcinoma

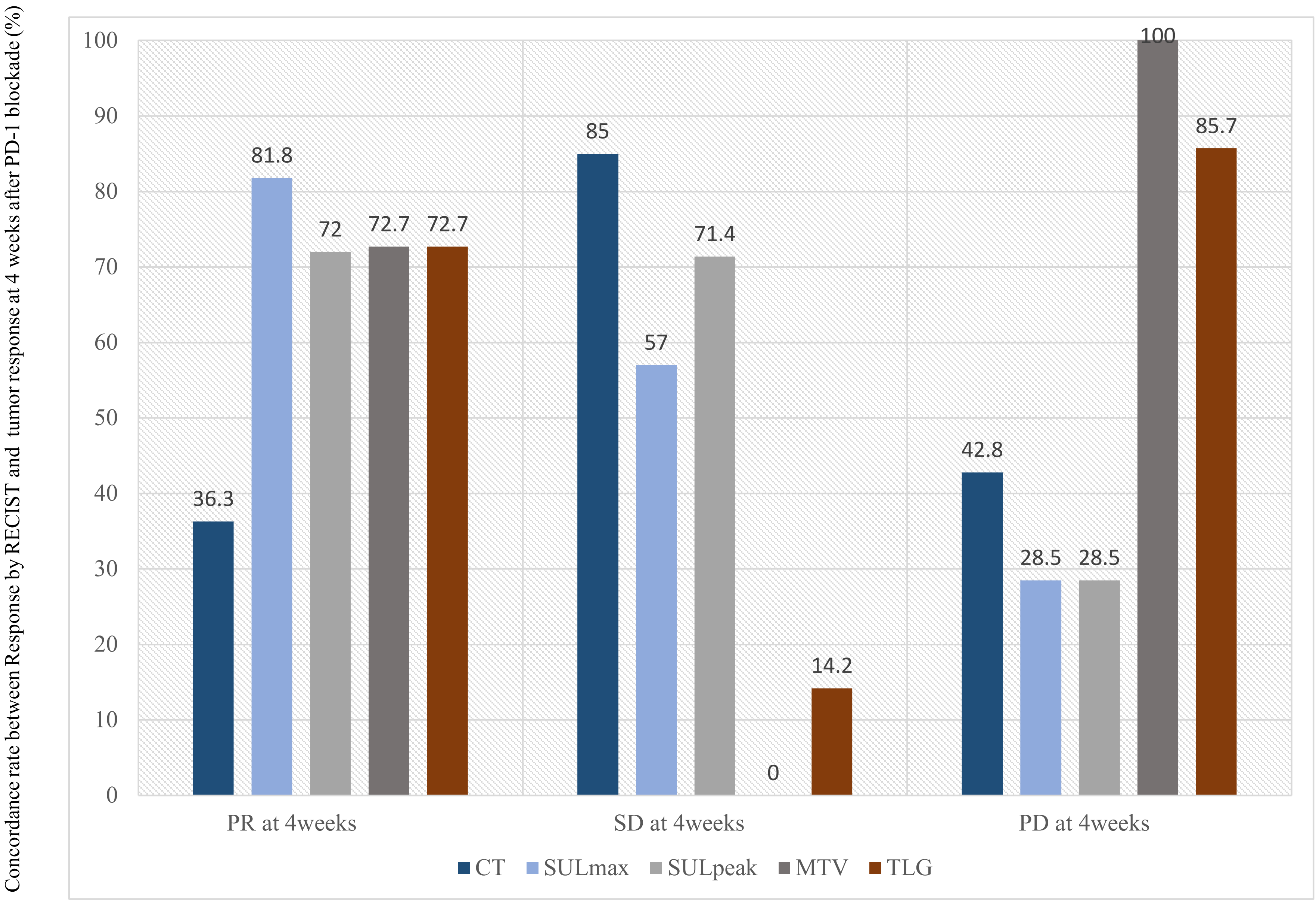

Figure C3

Concordance rate in CT and PET

Legends: concordance rate at 9 weeks in patients with non-adenocarcinoma

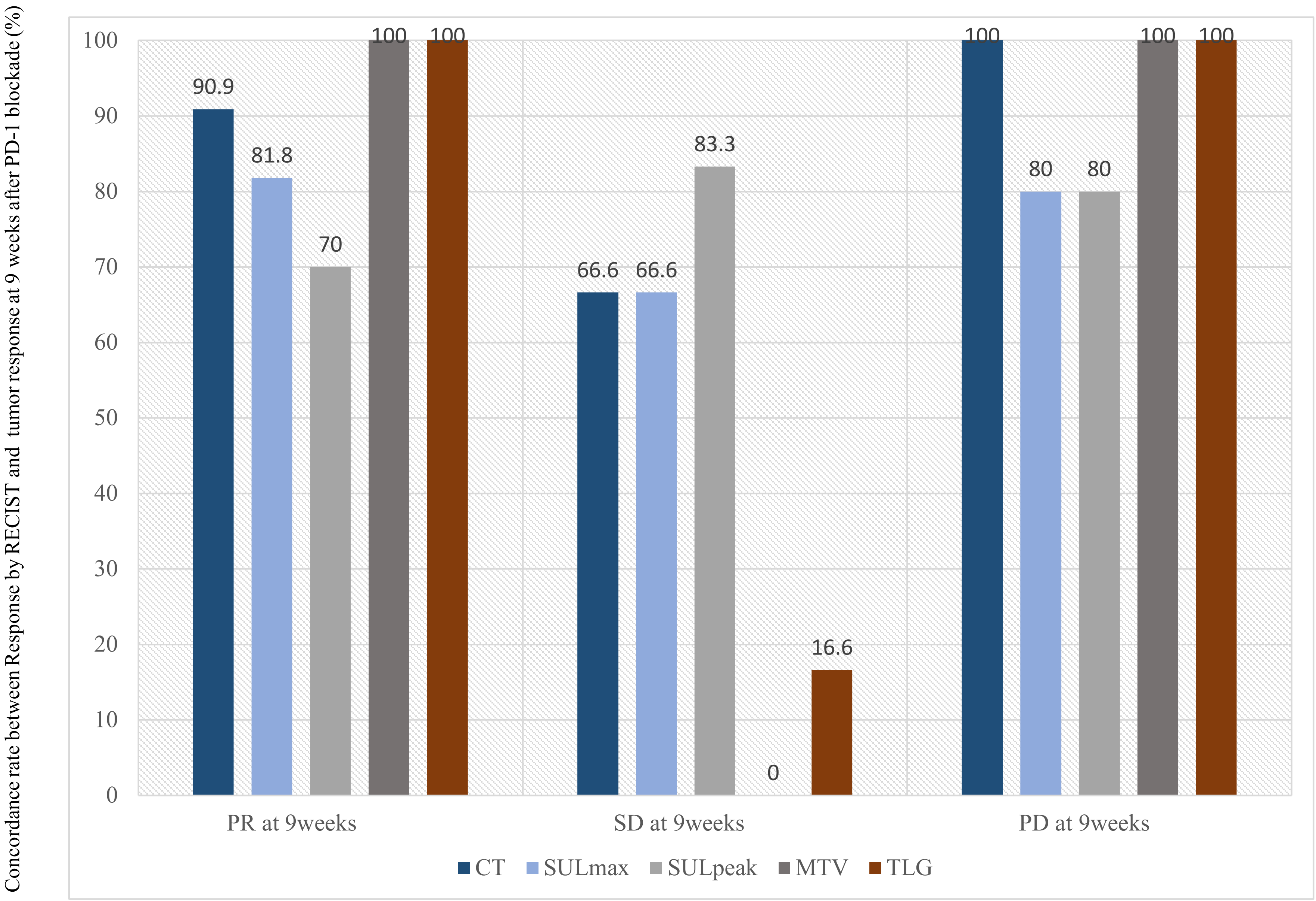

Figure C4

Concordance rate in CT and PET

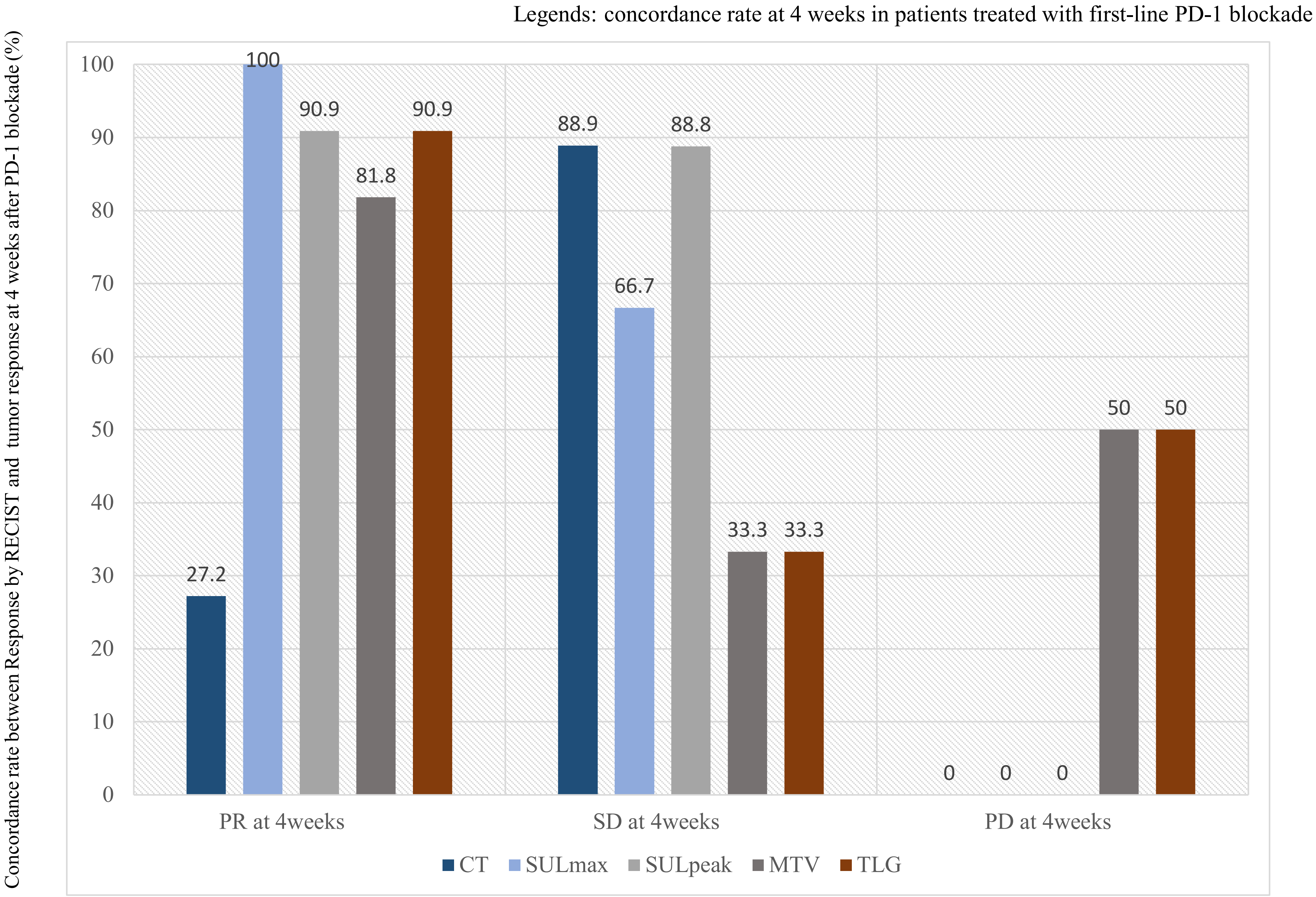

Figure C5

Concordance rate in CT and PET

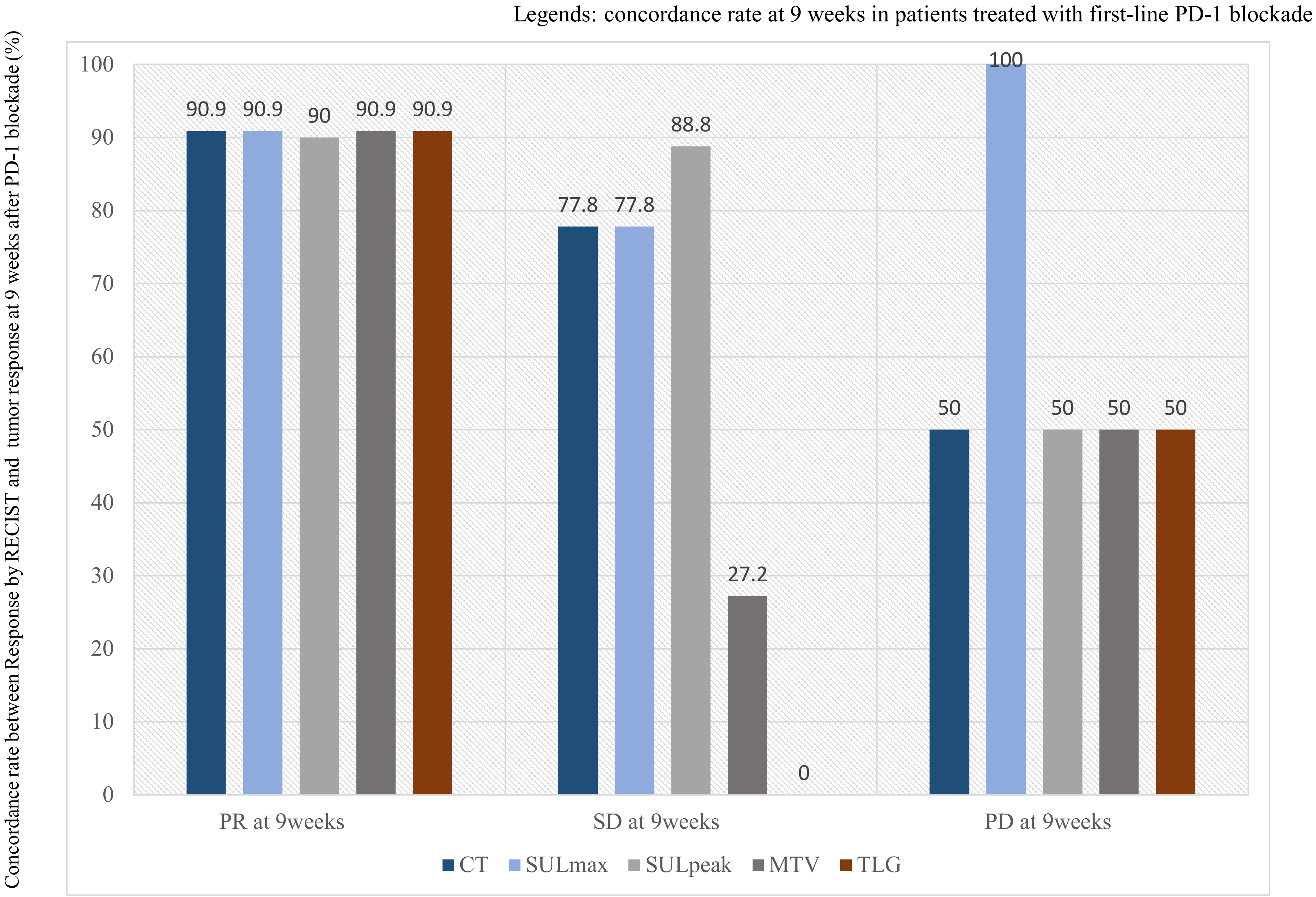

Figure C6

Concordance rate in CT and PET

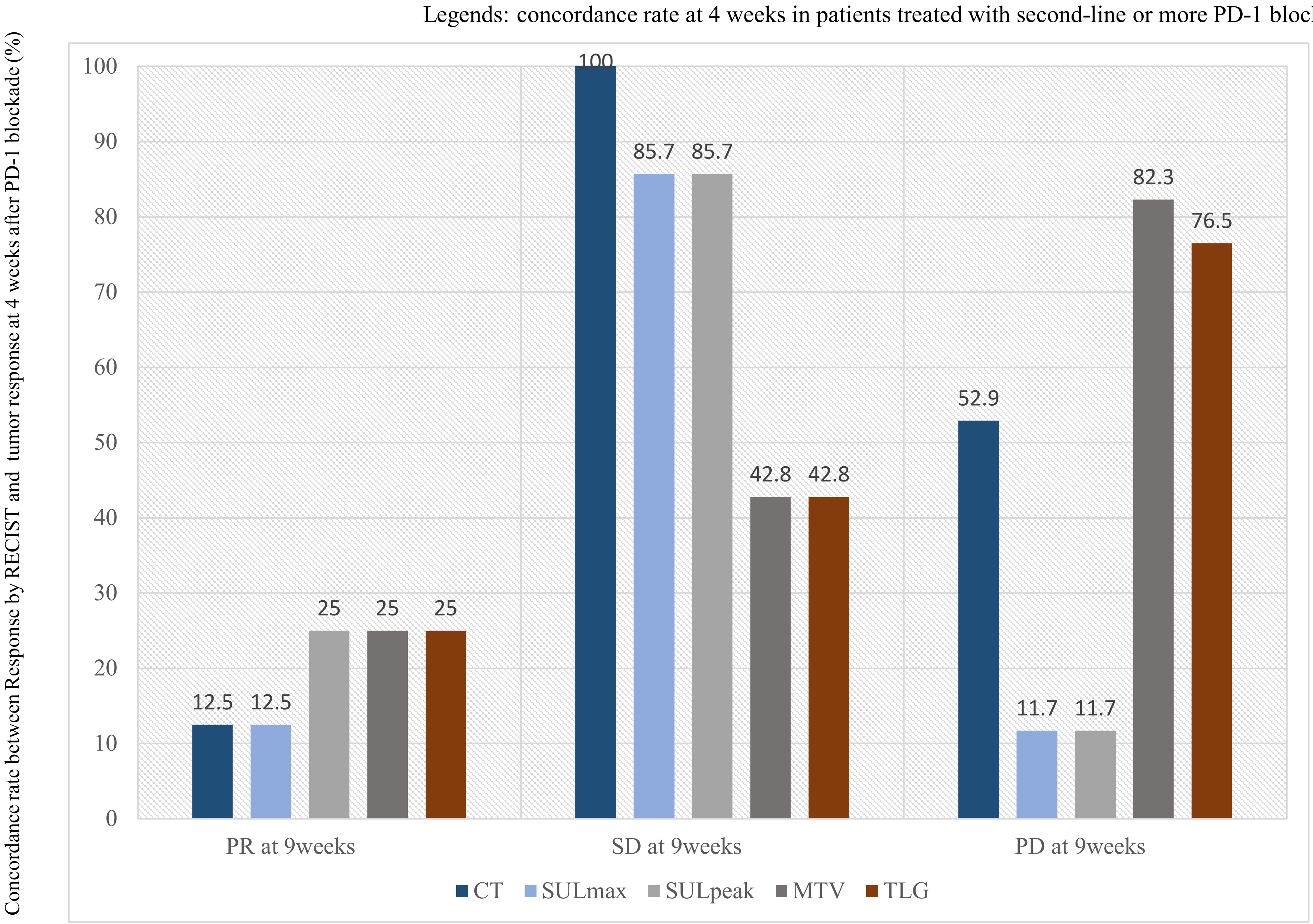

Figure C7

Concordance rate in CT and PET

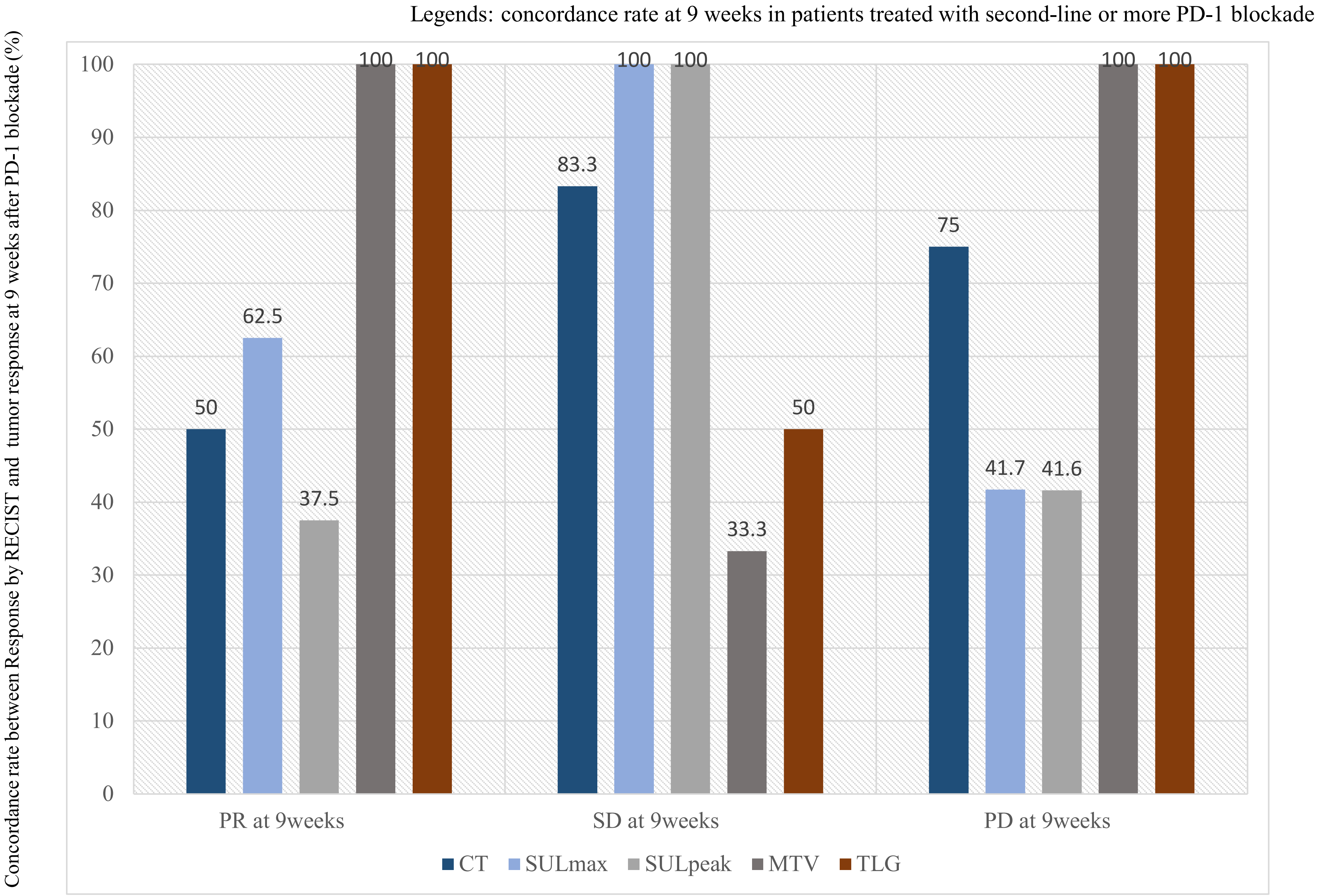

Figure C8
