# Supplementary material for: Prospective assessment using 18F-FDG PET/CT as a novel predictor for early response to PD-1 blockade in non-small-cell lung cancer
Source: Sci Rep. 2022 Jul 12;12:11832. doi: 10.1038/s41598-022-15964-3 (PMC9276827; doi:10.1038/s41598-022-15964-3)
Supplement: Supplementary file 3 — Supplementary Information 3. [file 41598_2022_15964_MOESM3_ESM.docx]

**Supplemental files**

**Figure A:**

Schedule of ^18^F-FDG PET/CT imaging in the present study:

^18^F-FDG PET/CT imaging at baseline is performed within 30 days of study registration. Second and third PETs are planned to be performed at 4 and 9 weeks after PD-1 blockade monotherapy.

**Figure B:**

Progression-free survival (PFS) (B**1**) and overall survival (OS) (**B2**) according to objective response confirmed based on RECIST.

The median PFS and OS of PR, SD, and PD were 435, 168, and 65 days, respectively (PR vs. SD, *p*=0.029; SD vs. PD, *p*=0.001), and not reached, 305, and 282 days, respectively (PR vs. SD, *p*=0.004; SD vs. PD, *p*=0.276).

**Figure C:**

Concordance rate between Response by RECIST and tumor response at 4 weeks and 9 weeks after PD-1 blockade according to treatment lines and histological types; concordance rate at 4 weeks (C1) and 9 weeks (C2) in patients with adenocarcinoma, that at 4 weeks (C3) and 9 weeks (C4) in those with non-adenocarcinoma, that at 4 weeks (C5) and 9 weeks (C6) in those treated with first-line PD-1 blockade, and that at 4 weeks (C7) and 9 weeks (C8) in those receiving second-line or more PD-1 blockade.
